# Rhesus monkeys metacognitively monitor memories of the order of events

**DOI:** 10.1038/s41598-018-30001-y

**Published:** 2018-08-01

**Authors:** Victoria L. Templer, Emily Kathryn Brown, Robert R. Hampton

**Affiliations:** 1 Department of Psychology, Providence College, 1 Cunningham Sq., Providence, RI 02918 USA; 2 Department of Psychology and Yerkes National Primate Research Center, Emory University, 201 Dowman Dr., Atlanta, GA 30322 USA

## Abstract

Human working memory is a capacity- and duration-limited system in which retention and manipulation of information is subject to metacognitive monitoring and control. At least some nonhuman animals appear to also monitor and control the contents of working memory, but only relatively simple cases where animals monitor or control the presence or absence of single memories have been studied. Here we combine a comparatively complex order memory task with methodology that assesses the capacity to introspect about memory. Monkeys observed sequential presentations of five images, and at test, reported which of two images from the list had appeared first during study. Concurrently, they chose to complete or avoid these tests on a trial-by-trial basis. Monkeys “knew when they knew” the correct response. They were less accurate discriminating images that had appeared close in time to one another during study and were more likely to avoid these difficult tests than they were to avoid easier tests. These results indicate that monkeys can metacognitively monitor relatively complex properties of the contents of working memory, including the quality of representations of temporal relations among images.

## Introduction

To most people, “memory” means information of which we are consciously aware. But memory consists of many types, distinct from one another neurobiologically, functionally, and phenomenologically^1,2^. It is likely that we are never conscious of the majority of our memories, despite the fact that they control our behavior. For example, people are often not consciously aware of grammatical rules despite correctly using them^3,4^. We learned and remember how to ride a bicycle, but it is difficult to explicitly explain how we ride. The contents of working memory are often considered to be the information to which we are attending consciously, and we are able to metacognitively monitor this information. e.g.^5,6^.

Comparative psychologists, evolutionary biologists, and cognitive neuroscientists are eager to determine the defining characteristics of the memory systems present in nonhuman animals. Knowing the extent to which humans and other animals share the same set of memory systems is critical to understanding the evolution of memory and to evaluating the validity of animal models of memory used in neurobiological research. Mapping the distribution of these memory systems among species will inform us about when in our evolutionary past each system appeared, and will provide data necessary to determine what selection pressures promote the emergence of these distinct memory systems^7,8^. Using particular behavioral assays as models of human memory will be useful only to the extent that these animal models capture both the biology and psychology of the memory systems they are intended to model. Homology with human biological systems is critical if biomedical model systems are to yield results relevant to human health.

Representation of the order in which events occurred is a critical function of memory. Memory for temporal order underlies our capacity to detect causation, to sequence complex action, and the autobiographical knowledge grounding our sense of self. These functions of memory often depend on cognitive control, which can be adaptively modified by feedback from cognitive monitoring^9,10^. Because the capacity for cognitive control correlates positively with general intelligence in humans^11^, documenting differences in the extent of cognitive control across species will advance understanding of the evolution of intelligence. Determining which types of memory are accessible to cognitive monitoring and sophisticated cognitive control is therefore an important part of the comparative study of intelligence. Here we assess the extent to which rhesus monkeys *(Macaca mulatta)* engage in introspective cognitive monitoring of memory for the order of events.

Monkeys, rats, and other animals remember the order in which events occurred. Monkeys shown a set of objects in sequence, then subjected to a brief delay, selected the object that occurred earlier in the sequence^12^. The ability of monkeys to correctly judge which object appeared earlier depended on the integrity of dorsolateral frontal cortex. In a similar task, rats were presented with a sequence of five odors and later were rewarded for selecting the odor that had occurred earlier in the study list. Rodents with either hippocampal, anterior thalamus, or medial prefrontal damage were impaired on order judgments but not on recognition tests, indicating that memory for unique sequences is dependent on these structures and is dissociable from recognition accuracy^13–16^.

**Figure 1:**
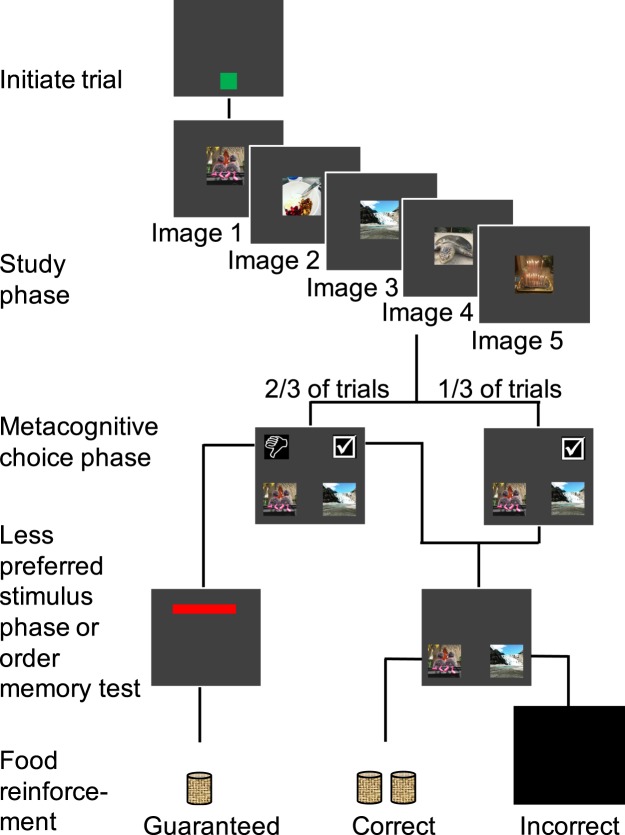
Test of memory for order with the decline-test option. After touching the green ready square to initiate a trial, monkeys saw and touched a list of five images in sequence. On two-thirds of trials, monkeys were given the option to accept or decline the memory test. If the test was accepted, monkeys were reinforced for selecting from two images the one that had occurred first in the study list. Images from the study list were not responsive to touch until the monkeys selected the *accept-test* symbol (check mark icon), after which monkeys could complete the memory test and receive a comparatively large reward of two pellets if correct. Errors resulted in no reward and a time-out during which the screen was blank. Selecting the *decline-test* option (thumbs down icon) resulted in escape from the test, followed by a smaller guaranteed reward of one pellet. On one-third of trials only the *accept-test* option, forcing monkeys to take the memory test.

We developed a visual test of memory for order for monkeys based on rodent tests of memory for the order in which odors were encountered^17^. Each trial, monkeys saw and touched a sequence of five images drawn randomly from a set of 6,000 images. At test, a randomly selected pair of images from the study list was presented, and monkeys were rewarded for selecting the image that had appeared earlier than the other in the study phase of the trial (Fig. 1). Thus, monkeys reported the order in which they had experienced events.

Previous reports of memory for the order of events in nonhuman animals provide no evidence regarding whether these memories are metacognitively monitorable, and subject to introspection. This is because there was no explicit response animals could make in these tests that is comparable to a human saying “I do not know” or “I do not remember.” However, techniques do exist that allow nonhuman animals to make responses that may be functionally equivalent to these human responses, essentially reporting whether or not they remember e.g.^17–22^. One implementation of these techniques consists of providing subjects a choice between taking a memory test for the possibility of a comparatively large reward if correct, or avoiding the memory test with the assurance of a guaranteed small reward e.g.^19,20,23,24^. Under these conditions, animals that can discriminate between remembering and not remembering should be more likely to choose to decline difficult tests and accept easy tests. Under a variety of testing conditions, monkeys do indeed selectively decline difficult memory tests, consistent with memory monitoring^19,23,25,26^. However, these tests of memory monitoring have been limited to memory for single items in isolation.

Here we test whether monkeys monitor a feature of memory substantially more complex than simply the presence or absence of memory. Accuracy in tests of memory for the order of events is governed, in part, by the number of events that intervened between the events being judged, such that the further two events are separated, the easier it is to correctly report which came first^13,17,27^. Thus, accuracy varies as a function of memory for the relation between events, in addition to memory for the individual events. By combining the order memory paradigm with the memory monitoring paradigm described above, we assessed whether monkeys are metacognitively sensitive to the reliability of their memory for the temporal relations between events. On *choice trials* monkeys chose between declining and taking memory tests, while on *forced trials* they only had the option of taking the test (Fig. 1). Forced test trials are like conventional tests of memory and provide a baseline measure of accuracy, allowing us to chart the difficulty of different trial types. Choice trials measure the degree to which monkeys are sensitive to subjective differences in memory across different trial types. If memory for the order of events in monkeys is accessible to monitoring, then monkeys should be more likely to decline more difficult tests than easier tests. Monkeys should also be more accurate, on average, on chosen than on forced trials if they are indeed selectively declining more difficult trials and accepting easier ones.

## Results and Discussion

**Figure 2:**
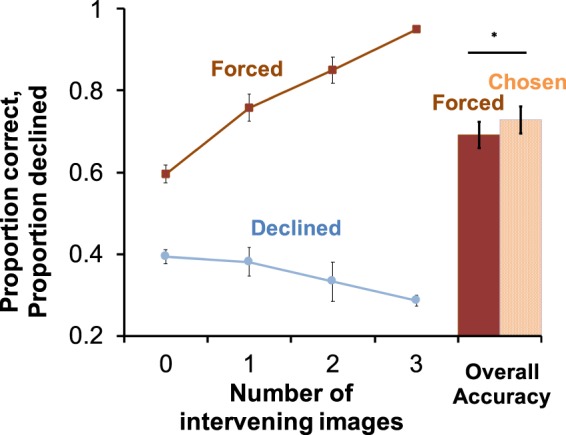
Accuracy and use of the decline-test response. Monkeys were more accurate the further separated the test images were in the study list (left, top line; F_3,15_ = 43.97, P < 0.001). Monkeys used the *decline-test* response in a pattern that mirrored accuracy, with monkeys more likely to decline difficult than easy trials (left, lower line; F_3,15_ = 11.06, P < 0.001). As a result of selective use of the *decline-test* response on more difficult trials, monkeys were more accurate on tests they chose to take than on those they were forced to take (right, bar graph; paired sample t-test t_5_ = 4.29, p < 0.01). Error bars represent standard error of the mean.

Monkeys selectively declined difficult trials, indicating that they “knew when they knew,” a capability that likely depends on the ability to monitor, or introspect about, memory. Monkeys performed well in the primary order memory task and were most accurate on trials in which many images intervened during study between the two images presented at test (Fig. 2, left, upper line). Mirroring this accuracy function, monkeys were more likely to avoid more difficult than easier memory tests (Fig. 2, left, lower line). Because use of the *decline-test* response tracks accuracy, and both vary according to the number of intervening images during study, these results show that monkeys are able to introspect about the reliability of their memory for the relations between events, in addition to their memory for individual events, as has been reported previously^18,20,23^. A consequence of monitoring memory in this way was that monkeys were more accurate overall on trials they chose to take than on trials they were forced to take (Fig. 2, right, bar graph). This pattern of behavior establishes a strong functional parallel between the behavior of monkeys and the human ability to monitor the contents of working memory.

Two classes of alternative explanations may limit the significance of these findings. First, use of the *decline-test* response may have been controlled by some publicly observable correlate of task difficulty, rather by than introspection about memory. Second, the order memory task we used may not depend on memory for the temporal relationships among images, *per se*, but instead be based on a continuous relative familiarity signal. We consider these alternatives below.

Human awareness of memory is private and introspective. We often “know that we know” because we subjectively experience memory, not because we are behaving effectively or quickly. What makes this monitoring subjective is that the owner of the memory has privileged access to the status of the memory. An observer can take note of the behavior of the rememberer, but they cannot access their introspection about memory. Establishing memory monitoring in nonhumans therefore requires ruling out external or public sources of stimulus control of the *decline-test* response. Monkeys should not decline tests only because these tests have specific observable characteristics, or only because the monkey detects a change in its own behavior, such as vacillation between test options or slow response time^25,26^. Many tests have been reported in the primate metacognition literature that evaluate explanations that do not involve introspection and the evidence is strong that in at least some cases monkeys attend to internal, subjective signals that reflect the quality of ongoing cognitive processing or the strength of memory e.g.^17,18,28^, While we cannot repeat all these tests of alternative explanations for apparent memory monitoring with the order memory paradigm reported here, we address two of the most likely alternative explanations: use of response latency as a discriminative cue and response competition.

When monkeys encounter a test for which they do not know the correct response, they may be slow to respond. Longer response latencies for tests where memory is poor could allow monkeys, and objective observers, to discriminate between trials on which the monkey does and does not know the answer. This alternative source of stimulus control for the *decline-test* response is only viable when accuracy and response latency are negatively correlated. Monkeys were indeed slower to select the *decline-test* than the *accept-test* response on average (mean decline = 1565 ms; mean accept = 1367 ms; F_1,5_ = 7.056, P = 0.045). However, these response latency differences did not vary with the number of intervening images (main effect of intervening images, F_3,15_ = 0.683, P = 0.576; choice X intervening images interaction: F_3,15_ = 1.60, P = 0.230). Thus, because the number of intervening images and response latency were not related, response latency cannot be the stimulus that controlled use of the *decline-test* response, which did vary as a function of intervening images. At the same time, it is likely that memory monitoring processes cause selection of the *decline-test* response to be slower than selection of the *accept-test* response overall^18,29^. Memory monitoring involves checking whether the needed information is present. Detecting the presence of such information takes less time than does determining that it is absent. If monitoring detects the target information, the monkey promptly selects the *accept-test* response. If monitoring does not immediately detect the relevant information, a slightly longer search ensues before the monkey selects the *decline-test* response if the information is not eventually detected.

In many situations, behavioral options may compete with one another. In some studies, the behavioral measure of memory monitoring has been put in direct competition with selecting one of the test responses e.g.^22,30^. In such circumstances the appearance of memory monitoring can emerge as a result of competition between behavioral options. If memory is strong, selecting the correct response may happen rapidly and exclude the possibility of selecting the *decline-test* option or engaging in other behaviors that might be interpreted as memory monitoring. In contrast, when memory is weak, selection of a test response is prolonged, creating more opportunity for selection of the *decline-test* option. As a result, selecting the *decline-test* option is more likely when memory is weak, even though this pattern may not result directly from monitoring memory. The design of this study makes such direct competition unlikely. Monkeys did see the memory test at the time they were choosing whether to accept or decline the test, but the test images were not responsive to touch until they had selected the *accept-test* option. Because the monkeys had to choose to accept or decline the memory test before they could complete it, there was no opportunity for selecting a test response to displace choice of the *decline-test* response.

Because memory fades over time, memory strength tends to correlate with order of presentation, with items presented earlier weakly represented compared to more recent items. Monkeys could potentially select the image that occurred earlier by identifying the image with the weaker memory strength. We directly tested this hypothesis in a previous study using this same order paradigm^17^. We presented monkeys with probe trials in which an image from the study list was presented with an image the monkeys had not seen before. The novel image should have the lower memory strength, and so according to the memory strength hypothesis, should be selected as having occurred earlier. Instead, monkeys reliably chose the image from the study list, indicating that choice was not controlled by memory strength.

While the work cited above shows that memory strength does not guide discrimination of order, memory strength might still influence use of the metacognitive *decline-test* response. If so, this might make the metacognitive monitoring reported here more akin to monitoring the strength of a single memory, as has already been demonstrated in previous tests of nonhuman metacognition, rather than monitoring of memory of the relation between remembered items. We therefore tested whether the presumed memory strength of the images making up a test pair influenced use of the *decline-test* response by sorting trials into categories determined by whether the pair of test images had occurred early or late in the study list. For example, a test with images one and two would be considered “early” and a test with images 4 and 5 would be considered “late.” We found no reliable relationship between early and late images and use of the decline test response, and therefore no evidence that memory strength controlled metacognitive responding (4 pairs with 0 intervening images: F_3,15_ = 0.511, P = 0.681; 3 pairs with 1 intervening image: F_2,10_ = 1.08, P = 0.376; 2 pairs with 3 intervening images 2: t_5_ = −2.55, P = 0.051).

The memory for order task used in the present study was modeled after an analogous task in which rats smelled five odors in sequence and then selected the odor that came earlier in the sequence after a three-minute delay^13,14^. The rodent version of this task is a prominent animal model of episodic memory, in large part because the memories depend on an intact hippocampus, as is also the case in humans, e.g.^31,32^. Monkeys in the current task similarly discriminated the order in which they had seen images, but their memory was only tested after very brief delays more consistent with working memory than long-term episodic memory. It would be of great interest to develop similar tests for monkeys that involve longer retention intervals and that test for additional features of episodic memory, such as binding with contextual information^33^. The present results indicate that monkeys encode information about the order in which events occurred in a way that is accessible to memory monitoring, but, because of the short retention intervals used, these results do not provide strong evidence for monitoring of episodic memory in monkeys.

Avoiding situations in which necessary knowledge is not available, as our monkeys demonstrated here, is probably only one of many functions of memory monitoring. Memory monitoring likely evolved at least in part because it provides feedback for cognitive control processes. Memory monitoring may provide information about whether working memories are fading and in need of refreshing^34,35^, whether target information is being retrieved from memory successfully, and whether studying material or searching for information has yielded sufficient results^36^. Because the capacity for cognitive control correlates positively with general intelligence in humans, e.g.^37^, documenting differences in the extent of cognitive control is likely critical for understanding the different intelligences found among species. Determining which types of memory are accessible to cognitive monitoring and control is therefore an important part of the comparative study of intelligence. We found that memory for order is accessible to memory monitoring in monkeys. This discovery of monitoring of memory for order is one piece of a larger effort that should involve direct quantitative comparisons among species of the capacity for cognitive monitoring and cognitive control. Documenting such differences may provide one account for differences in animal intelligence.

## Methods

### Subjects

We used six six-year-old male rhesus monkeys *(Macaca mulatta*) that had approximately two-years computer testing experience, including previous participation in metacognition experiments^18,38^. Monkeys had already been trained to discriminate the order of events^17^.

### Apparatus and Materials

Monkeys were tested in their home cages on computerized touch-screen test systems that were attached to the front of each cage. Each test system consisted of a 15-inch LCD color monitor (3 M, St. Paul, MN or Elo, Milpitas, CA) running at a resolution of 1024 × 768 pixels, stereo speakers, two automated food dispensers (Med Associates Inc., St. Albans, VT), and two food cups below the screen. Food reinforcement consisted of 94 or 97 mg nutritionally complete primate pellets (Bio-Serv, Frenchtown, NJ and Purina TestDiet, Richmond, IN). We presented stimuli and collected responses using programs written in Presentation (Neurobehavioral Systems, Albany, CA).

Six thousand color photographs collected from public online digital image databases were used as memoranda. Images of primates were not used. Images were resized to 300 × 300 pixels. The same stimuli had been used when monkeys originally learned the order task alone^17^.

### Monkey housing and testing conditions

Monkeys were pair-housed and kept on a 12:12 light: dark cycle with light onset at 7:00 am. Water was available *ad libitum*. All monkeys were fed a full ration of food at the end of testing each day. One to five test sessions (depending on the individuals’ work-pace) were conducted every other day between 10 am and 5 pm six days per week.

Each monkey had access to his cage-mate at all times except during testing and during feeding at the end of the day. While testing, monkeys were separated by insertion of plastic dividers between cage-mates that allowed limited visual and physical contact, but prevented access to the cage-mate’s testing equipment. Testing systems were locked to the front of each monkey’s cage and cage doors were raised giving subjects full access to the screen during testing.

### Refamiliarization with metacognitive MTS task

Monkeys were presented with a delayed match-to-sample (DMTS) task with a concurrently presented *decline-test* option, as described in^23^. To ensure that monkeys remembered how to use the *decline-test* response adaptively, they were required to demonstrate a 30% difference in percent of trials declined between the easiest and most difficult conditions.

### Order task

After it was determined that monkeys continued to monitor memory in the DMTS task, subjects were re-familiarized with the order task. Monkeys first received four sessions of the order task they had previously learned^17^. A green square appeared at the bottom of the screen and remained until the monkey touched it to start a trial, after which a photograph appeared in the middle of the screen on a gray background (Fig. 1). To ensure that the monkey saw the image, the image was only sensitive to touch (FR 2) after a required minimum study period of 250 milliseconds. After the image was touched twice, a 500 millisecond inter-stimulus interval (ISI) occurred during which the screen was gray, and a second randomly selected image appeared in the same place as the first. After five sample images were presented and touched, a 500-millisecond retention interval occurred and then two test images from the list appeared to the left and right of the bottom of the screen separated by 400 pixels.

Selection of the image that had occurred earlier in the study sequence was rewarded with a positive auditory stimulus and a food pellet 100% of the time. Selection of the image that had occurred later in the sequence was followed by a negative auditory stimulus and a 10 second time-out during which the screen was black. The position of the correct and incorrect test images was pseudo-randomized such that the correct image did not appear on the same side of the screen for more than four consecutive trials, and the correct image appeared in each location equally often. Trials were separated by a 500 millisecond inter-trial interval (ITI) during which the screen was black. Tests consisted of images from each of the ten possible list position pairs, which included adjacent (e.g., 1 vs. 2) and non-adjacent images (e.g., 3 vs. 5). Each type of test was presented 12 times in a session, resulting in 120-trial sessions.

All parameters were as described in our previous work^17^, with the exception that the two test stimuli were presented at the bottom of the screen rather than the middle of screen. It was necessary to lower the locations of two primary test stimuli so that the *decline-test* and *accept-test* symbols could be in the same locations subjects had experienced on previous tasks^23^. Subjects received four sessions, after which accuracy on forced trials fell within the 60–80% range. Accuracy in this range ensured that monkeys experienced both trials in which they knew which image appeared earlier in the sequence and trials in which they did not. Occurrence of trials when monkeys have remembered and when they have forgotten is critical because it means that if subjects accurately judge the strength of their own memories, and decline trials in which memory is poor, they will experience and overall increase in rewards.

### Refamiliarization with metacognitive perceptual task

Monkeys were then given another *decline-test* task on which they had previously demonstrated cognitive monitoring. Monkeys were required to discriminate arc length and were offered a *decline-test* response on some trials. Subjects received at least one session of this test as described in^23^ to further ensure generalized adaptive use of the *decline-test* response. Monkeys were required to demonstrate a 30% difference in percent of trials declined between the easiest and most difficult conditions.

### Refamiliarization with order task

Monkeys were given one more session of the order task to ensure that accuracy remained between 60–80%.

### Decline-test option in the order task

All task parameters were the same as described above, except that the *decline-test* and *accept-test* options were added. On one-third of tests, monkeys received forced tests in which the *decline-test* response was not present and in order to proceed to the order discrimination the *accept-test* option must be selected. On two-thirds of trials monkeys received choice tests in which both the *accept-test* (check) and *decline-test* (thumbs down) options were available (Fig. 1). If the test was declined, a black screen with a red bar at the top of the screen appeared. Touching the red bar was rewarded with one food pellet. The number of touches required to receive a reward for touching the red bar was individually-titrated to avoid floor and ceiling use of the *decline-test* response^23^. This titration took place before the sessions described here. For monkeys that previously demonstrated floor and ceiling use, if they did not use it often enough, the FR was halved; if they used it too often, the FR was doubled. We required that these monkeys use the *decline-test* response on at least 30% of trials, and on no more than 70% of trials. This ensured that monkeys used the decline-test response at a rate that permitted measurement of both increases and decreases in the use of this response across trials of different difficulty. The FR was not changed during the testing described here. If the *accept-test* symbol was selected, the *accept-* and *decline-test* images disappeared, and the two images from the study list became sensitive to touch. If monkeys selected the test image that had occurred earlier during study, two food pellets were delivered along with the same auditory reinforcement as used earlier. Correct responses on forced trials were also rewarded with two pellets. The number of forced and choice trials was distributed equally across the 10 list positions such that within one 120-trial session each of the 10 trial types occurred 4 times for forced trials and 8 times for choice trials. To obtain an accurate measure of accuracy with 20 unique trial types, monkeys received 20 sessions.

### Pretesting

All subjects displayed at least a 30% accuracy difference on forced vs. chosen trials on the both the MTS and arc discrimination tasks within one session. Averaging across the four sessions of the order task subjects received, the mean accuracy was 68% correct. All subjects fell within the range of 60–80%.

### Statistical analyses

Proportions were arcsine transformed before statistical analysis to better approximate the normality assumption underlying parametric statistics^39^^, p.155^. Latencies analyzed were medians for correct trials only. T-tests were two-tailed. An alpha level of p < 0.05 was applied to all analyses. The Geisser-Greenhouse correction was used in cases in which the sphericity assumption was violated^39^ and the corrected degrees of freedom and p values are reported (statistical analysis performed in SPSS, IBM, version 24, Chicago, IL).

### Data availability

The datasets generated during and/or analyzed during the current study are available from the corresponding author on reasonable request.

### Ethics statement

All procedures carried out in this study were approved by the Institutional Care and Use Committee (IACUC) of Emory University which were performed in accordance with the National Institutes of Health Guide for the Care and Use of Laboratory Animals.
